# Lateral gene transfers and the origins of the eukaryote proteome: a view from microbial parasites

**DOI:** 10.1016/j.mib.2014.11.018

**Published:** 2015-02

**Authors:** Robert P Hirt, Cecilia Alsmark, T Martin Embley

**Affiliations:** 1 Institute for Cell and Molecular Biosciences, Faculty of Medical Sciences, Newcastle University, Newcastle upon Tyne, NE2 4HH, UK; 2 Division of Pharmacognosy, Department of Medicinal Chemistry, Uppsala University, Biomedical Center, S-751 23 Uppsala, Sweden; 3 Department of Virology, Immunobiology and Parasitology, National Veterinary Institute, Uppsala, Sweden

## Abstract

Our knowledge of the extent and functional impact of lateral gene transfer (LGT) from prokaryotes to eukaryotes, outside of endosymbiosis, is still rather limited. Here we review the recent literature, focusing mainly on microbial parasites, indicating that LGT from diverse prokaryotes has played a significant role in the evolution of a number of lineages, and by extension throughout eukaryotic evolution. As might be expected, taxonomic biases for donor prokaryotes indicate that shared habitat is a major factor driving transfers. The LGTs identified predominantly affect enzymes from metabolic pathways, but over a third of LGT are genes for putative proteins of unknown function. Finally, we discuss the difficulties in analysing LGT among eukaryotes and suggest that high-throughput methodologies integrating different approaches are needed to achieve a more global understanding of the importance of LGT in eukaryotic evolution.

## Highlights

• Prokaryotic LGT to microbial parasites is a dynamic and on-going process.
• Identified LGTs are mainly involved in metabolic pathways.
• Both LGT and EGT of prokaryotic origins are contributing genes to eukaryotic genomes.
• Integrating different methodologies is needed to truly recognise the extent of LGT affecting eukaryotes.

**Current Opinion in Microbiology** 2015, **23**:155–162

This review comes from a themed issue on **Genomics**

Edited by **Neil Hall** and **Jay CD Hinton**

For a complete overview see the Issue and the Editorial

Available online 5th December 2014

**http://dx.doi.org/10.1016/j.mib.2014.11.018**

1369-5274/© 2014 The Authors. Published by Elsevier Ltd. This is an open access article under the CC BY license (http://creativecommons.org/licenses/by/3.0/).

## Introduction

Novel genes derived from a number of processes; including gene duplications, *de novo* gene formation, and LGT; contribute to genomic and phenotypic plasticity and can drive adaptive evolution [1]. LGT in prokaryotes is recognised to play a major role in providing novel protein coding genes and contributing adaptive traits, including the archetypical resistance to antibiotics [2]. The frequency and origins of LGT among eukaryotes and its impact on their biology is still relatively poorly understood [3] but is also increasingly recognised as a significant source of novel genes [4, 5]. Compared to prokaryotes identifying LGT in eukaryotes is more difficult due to the confounding effect of their (i) complex origins involving at least two prokaryotic lineages, (ii) more complex genome architecture and protein coding capacities, (iii) sparse and biased taxonomic sampling of genome sequence data and (iv) lack of phylogenetic resolution for the major eukaryotic lineages [6]. These factors, along with the intrinsic difficulties of inferring single gene phylogenies, render annotations and evolutionary inferences of eukaryotic protein coding genes often less reliable and more sensitive to sequence database taxa sampling and to different parameters of evolutionary models in bioinformatic tools [6].

Protein coding genes in eukaryote nuclear genomes are currently thought to have originated from DNA from at least two distinct prokaryotic lineages, an archaeal source, thought to represent the original host that evolved into a nucleated cell and an alpha-proteobacterial endosymbiont that eventually evolved into mitochondria [6, 7]. Additional nuclear genes of bacterial origin can be identified among eukaryotes possessing plastids, derived from a cyanobacterial primary endosymbiont or from secondary/tertiary endosymbioses involving eukaryotic endosymbionts with primary/secondary plastids [7, 8]. Eukaryotic nuclear genes derived from endosymbionts are defined as endosymbiotic gene transfers (EGT) [7], which for convenience we differentiate here from LGT from other sources. Mobile genetic elements, including viruses and transposable elements, can also be integrated into nuclear genomes [1, 9, 10]. We shall focus here on eukaryotic genes of prokaryotic origins in microbial parasites and discuss how these data are pertinent to the question of the relative contribution of prokaryotic LGT during eukaryote diversification more generally. Notably, in a given eukaryotic genome the number of genes of bacterial origin are typically more numerous (∼2/1 ratio across 14 genomes analysed in [11]) and significantly more variable than those that can be traced to an archaeal origin, highlighting the higher evolutionary plasticity of the former [11]. The growing list of LGT identified from various prokaryotic donor lineages in different eukaryotic lineages suggests that LGT has played a significant role in shaping eukaryote protein coding capacity throughout eukaryote diversification [12^•^].

## Parasites as model systems to study LGT in eukaryotes

Parasitic microbial eukaryotes have dramatic impact on the health of humans, farmed animals and plants, in addition to wildlife [13, 14•]. They also represent important model systems to study the evolution of eukaryotic cells and genomes as they are dispersed across eukaryote diversity [15]. The number of genome sequences from eukaryotes is increasing rapidly although sampling is still rather biased towards animals, fungi, plants and their parasites [16]. At a finer evolutionary scale sampling of genomes from different strains of a given species and closely related species represent an important source of data to investigate patterns of LGT acquisitions and losses and to study their potential link with phenotypic diversity and adaptions [2, 3].

We have recently investigated the genomes of 12 microbial parasites infecting humans and animals [12^•^] (Table 1 lists some examples), which include members of four of the currently recognised five eukaryotic super-groups [15]. For comparison we also included the free-living soil amoeba *Dictyostelium discoideum* [12] and list recently published data for additional free-living species in supplementary Table S1. Our analyses represent one of the broadest and most detailed investigations of relatively recent LGT, explicitly excluding EGT [12^•^]. This is pertinent, as numerous publications have reported eukaryotic LGT for small sets of genes or individual genomes using a range of different methodologies and selection criteria to identify candidate LGTs. This makes meaningful comparison of data between publications rather difficult. Indeed very different counts of LGT have been published for a given genome depending on the methodology and database used (Table 1 and supplementary Table S1) [12^•^].

## Animal hosts as a bazaar for LGT and dynamics of transfer

Animal microbial parasites have specialised for infecting different tissues in a given host including extracellular and intracellular niches [13]. Some are restricted to mucosal surfaces (e.g. *Trichomonas*), others are dependent on arthropod vectors (e.g. *Trypanosoma*) and enter their vertebrate hosts through a bite to initiate infections in the skin and/or in internal tissues. Mucosal and skin surfaces of humans and other vertebrates are hosts of a diverse and abundant microbiota comprising Bacteria, Archaea, microbial eukaryotes and viruses that are increasingly recognised as playing myriad roles in host biology [17^••^]. LGT among the bacterial microbiota of the gut mucosa was shown to be quantitatively more important (∼25× times) than among prokaryotes from other environments [18], hence the gut microbiota has been dubbed a bazaar for gene exchange [19]. Mucosal parasites interact with the highly abundant and dense vertebrate microbiota and for parasites dependent on vectors there is close contact with the microbiota of the arthropod digestive tract [20].

Our dataset comprised a mix of intracellular and extracellular, mucosal-dependent and vector-dependent parasites (Table 1), which provides opportunities to compare parasite life style and mode of transmission on the abundance and sources of LGTs. Our phylogenies identified relatively recent LGT from prokaryotic sources affecting all of the considered species (Table 1 and supplementary Table S1 — for methodology see [12^•^]). The fraction of identified LGT varied between 0.16% and 0.96% of protein coding genes per genome, rather smaller proportions compared to some reported LGT counts among prokaryotes [3, 21]. The smallest numbers of prokaryotic LGT were identified among the obligate intracellular parasites *Encephalitozoon cuniculi* (1 case) and *Cryptosporidium parvum* (8 cases) possibly due to the additional barrier of the host plasma membrane reducing access to bacterial DNA (Table 1). Notably, the microsporidian *E. cuniculi* has the lowest number of LGT and avoids all direct interaction with the outside world during its life cycle [22]. Mucosal (range 15–134 LGTs per genome, extracellular species) and vector-dependent parasites (range 16–63 LGTs per genome) (Table 1) and the free-living *D. discoideum* (60 LGTs, supplementary Table S1), experienced overlapping values of LGT counts indicating that these different life styles are all conducive to LGT.

**Figure 1:**
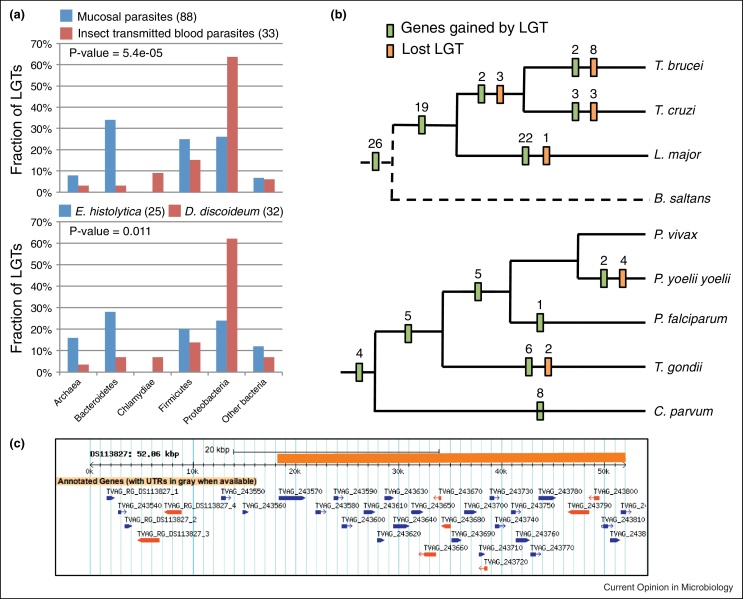
Candidate LGT among parasitic microbial eukaryotes. **(a)** Taxonomy of donor lineages for candidate LGTs. Comparison of the prokaryotic lineages inferred to be donating genes to the extracellular mucosal parasites *Entamoeba histolytica*, *Trichomonas vaginalis*, and *Giardia lamblia* compared with the inferred donor lineages for the insect-transmitted blood parasites *Trypanosoma brucei*, *T. cruzi*, *Plasmodium falciparum*, *P. vivax* and *P. yoelii yoelii* (top panel). Comparison of the prokaryotic lineages inferred to be donating genes to the parasite *E. histolytica* and its free-living amoebozoan relative *D. discoideum* (bottom panel). ‘Other bacteria’ comprise the Actinobacteria, Aquificae, Fusobacteria, Plantomycetes, Spirochaetes, or Tenericutes. Fisher's exact test was performed to test the null hypothesis that the taxonomy of the donors is distributed equally between the compared taxa. The *P*-values for the tests are indicated; they both reject the null hypothesis. The numbers of LGTs considered for each set of taxa are indicated between brackets. **(b)** Assessment of gains and losses of lateral gene transfer (LGTs) during parasite speciation. Maximum parsimony was used to map candidate LGTs on the species trees for taxa among (a) Trypanosomatidae and (b) Apicomplexa. Gains and losses are indicated as green and orange bars respectively. Characters were analysed using Dollo parsimony, so each character is allowed to have only a single gain, but may have multiple losses. It is inferred that 45 LGTs occurred (over 75 genes affected by LGT) before the divergence of the three parasitic Trypanosomatidae lineages. Interestingly, we detected 26 of the same LGTs in the genome of the free-living kinetoplastid *Bodo saltans* [45] using Blast similarity scores, suggesting these transfers may predate the transition to parasitism. Figures in panel (a) and (b) are derived from [12^•^]. **(c)** The mapping of annotated genes (red and blue genes indicate the differential orientation of the inferred open reading frames) on the scaffold DS113827 from the genome sequence data of *T. vaginalis* strain G3 [46]. A 32 kbp fragment (orange bar) was shown to be highly similar to the Firmicutes *Peptoniphilus harei* and encode 27 annotated genes. A matching gene cluster was found in all four additional investigated strains of the parasite [24]. Entries labelled with RG in their locus tags correspond to highly repetitive gene families, which are known to litter the genome of *T. vaginalis* [46]. The figure in panel c was generated using TrichDB [47].

Contrasting the pooled LGTs of the extracellular mucosal parasites to those of the insect-transmitted blood parasites indicated a significant bias towards the Bacteroidetes and Firmicutes for the donor lineages among the former (Figure 1a). This is consistent with gene sharing at mucosal surfaces of the digestive tract where these two bacterial lineages are known to represent the bulk of the biomass and taxonomic diversity [17^••^]. Similarly when contrasting the candidate donor lineages between the gut parasite *E. histolytica* and the free-living *D. discoideum* the former was also enriched for Bacteroidetes and Firmicutes donors reflecting the different habitats for the two Amoebozoa (Figure 1a). A few cases of candidate LGT from Eukaryotes to prokaryotes and/or eukaryote to eukaryote were also identified supporting LGT between mucosal species [12^•^]. More recent analyses of LGTs for several *Entamoeba* spp. have further highlighted gene sharing between mucosal parasites by strongly supporting a number of LGTs between *Entamoeba* and *Trichomonas* [23^•^]. This suggests that mucosal extracellular parasites are gaining bacterial genes in the same bazar as mucosal bacteria and can also contribute LGTs as donors.

Consistent with the taxonomic profile of prokaryotic donors sharing the same habitat as the parasites, a very recent candidate LGT in *Trichomonas vaginalis* was demonstrated to be shared between five clinical strains but absent from closely related *Trichomonas* species [24]. The 34 kbp fragment of bacterial origin encodes 27 annotated genes (Figure 1c) that are highly similar to sequences from the Firmicute *Peptoniphilus harei*, which can be isolated from patients with bacterial vaginosis (BV) [24], a condition also associated with infections by *T. vaginalis* [25]. The scaffold encompassing this large DNA fragment also includes several indigenous *T. vaginalis* genes (Figure 1c). The chimeric nature of this scaffold is consistent with integration of the bacterial DNA into the parasite's genome. Comparing the *Peptoniphilus sp.* derived genes between *T. vaginalis* strains indicated that different subsets of genes have undergone pseudogenisation [24]. These observations are consistent with a very recent LGT within the *T. vaginalis* lineage while infecting the human urogenital tract. Intriguingly LGTs from Bacteroidetes donors to *T. vaginalis* are in 89% of cases inferred to be derived from *Bacteroides* species [12^•^], a common genus in the gut of humans and other vertebrates [17^••^]. However the Bacteroidetes associated with the human female urogenital tract, in particular during BV, are typically from *Prevotella* and not *Bacteroides* species [25]. This suggests that an ancestor of *T. vaginalis* that was a gut parasite acquired these LGT from *Bacteroides* donors. This hypothesis can be tested by investigating the distribution of *Bacteroides* derived LGT across a range of *Trichomonas* species, all from the digestive tract — for example, *Trichomonas stableri* infecting the gut of birds and closely related to *T. vaginalis* [26].

Mapping LGT onto species phylogenies of sampled apicomplexan and kinetoplastid genomes respectively allowed us to gain insights into the process of LGT in relation to speciation of these parasites (Figure 1b). A total of 45 LGT were acquired by an ancestor to the three sampled kinetoplastids, compared to only 4 among the 5 apicomplexans. A number of LGTs are specific to, and some were lost by, a given lineage (Figure 1b). These data illustrate the highly dynamic nature of gene acquisition and loss during evolution of these groups. Those LGTs that have been retained during speciation are likely to be functionally important for the parasites.

## Functions of identified LGT: mainly metabolism and unknown functions

The majority of the identified LGTs were annotated as enzymes (62%), with 75% of them mapping onto the 11 major KEGG metabolic pathways particularly affecting amino acid and sugar metabolism [12^•^]. This pattern is consistent with the complexity hypothesis, put forward from the analysis of prokaryotic genomes, where operational (e.g. metabolism) genes are more likely to undergo LGT than informational (e.g. translation) genes [27]. Thirty five % of all the LGTs corresponded to genes with unknown functions, highlighting important gaps in our knowledge of the importance of the genes shared between bacteria and parasites [12^•^].

**Figure 2:**
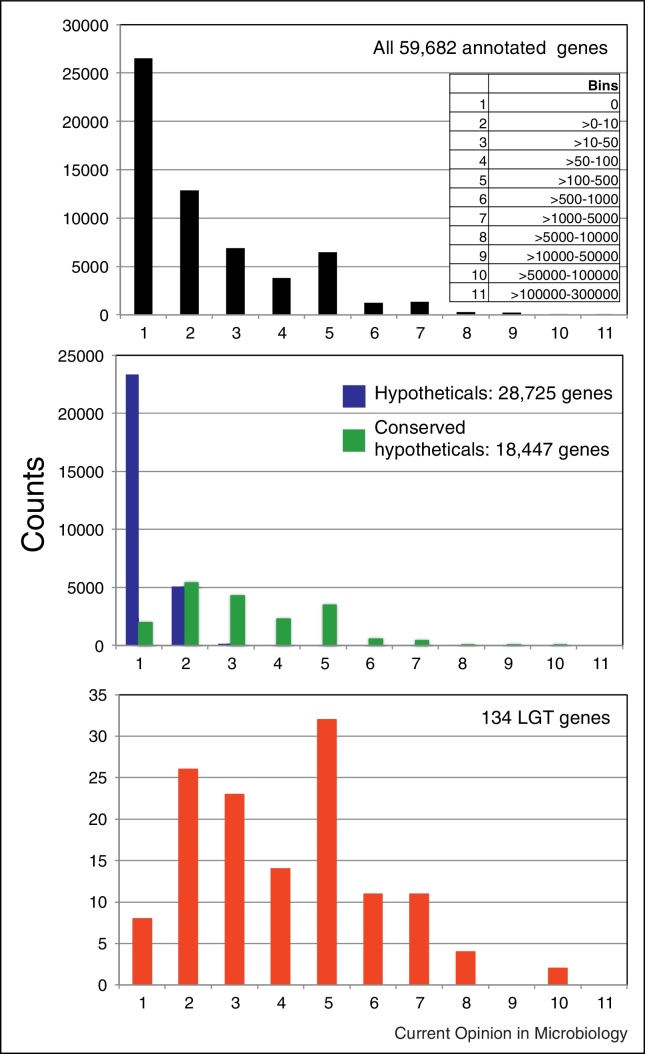
Evidence for transcription of LGT in *Trichomonas vaginalis*. The level of transcription of different gene sets was contrasted through histograms with 11 bins reflecting no read (bin 1, zero mean 3′-normalised reads across 11 conditions in [28]) to the highest level of transcription (bin 11, >100,000 to 300,000 mean reads). The inset in the top panel shows all bins with different levels of transcription expressed as the mean of 3-‘normalised reads across 11 tested growth conditions. The top panel illustrates the variation in transcription level between all annotated protein coding genes from the *T. vaginalis* G3 genome sequence data [46]. The middle panel contrasts protein-coding genes annotated as ‘hypotheticals’ (blue bars, with no BlastP hits in databases) versus ‘hypothetical conserved’ (green bars, with BlastP hits in databases). The bottom panel indicates the level of transcription of all LGT cases identified in [12^•^]. The LGT genes are notably skewed towards the right hand side of the histogram (higher level of transcription) compared to ‘hypotheticals’. This suggests that the majority of LGTs are likely to be functionally integrated into the biology of the parasite whereas the great majority of ‘hypotheticals’ are not and might represent pseudogenes or miss-annotations of spurious genes.

To extend to which LGTs are functionally integrated in the workings of the cell is often unknown [12^•^]. Hence the adaptive value of LGTs are typically inferred rather than demonstrated experimentally [5, 14•]. Transcriptomics can provide insight into this question by demonstrating whether an LGT is expressed and at what level compared to indigenous genes. Moreover correlation of expression with specific growth conditions might provide initial evidence for the adaptive value of a given gene. Interestingly, none of the 27 genes recently transferred to *T. vaginalis* from a *Peptoniphilus* species (Figure 1c) were transcribed at significant levels under different growth conditions in two distinct strains of the parasite [28, 29] (Table 2). By contrast, the majority of *T. vaginalis* candidates LGTs we identified [12^•^] have substantial levels of transcription (Figure 2, Table 2). In particular several enzymes gained through LGT mediating amino acid metabolism are up-regulated under glucose-restricted growth conditions, consistent with their involvement in energy production via amino acid catabolism [29]. Among nine identified LGT encoding enzymes potentially involved in host glycan degradation [12^•^], seven were expressed but two entries had no evidence for transcription (Table 2). Upon further investigation these two appear to represent a potential contaminant (TVAG_593180) and a pseudogene (TVAG_123020) (Table 2). For TVAG_123020 we could identify a close homologue, TVAG_371840, corresponding to a full-length gene that is transcribed (Table 2).

## Methodological considerations

Phylogenies probably still represent the gold standard for identifying LGT [30, 31]. However the inherent difficulties (biological and computational) in generating informative trees (selection of homologues, multiple sequence alignment, and tree inference) has motivated the development of surrogate or parametric methods that take advantage of blast hit lists or sequence composition anomalies [30, 31, 32]. The plethora of methods used across studies makes comparisons of the number of inferred LGTs between analyses rather difficult as different methodologies often identify different LGTs [32]. With the enormous increase in genome sequence data there is also a need to develop methodologies that scale with the increasingly large database [33^•^]. Another important limitation of classic phylogenomic approaches is that they are biased towards proteins for which meaningful alignments can be obtained; mainly relatively long proteins with simple domain organisation. For relatively short proteins and/or those with complex domain organisation, phylogenetics is difficult to implement and often lacks sufficient resolution, in particular within the framework of automated approaches required for larger datasets where manual curation is not feasible. Hence for a number of functionally important proteins, such as surface proteins in parasites, which includes some strong candidate LGTs supported by detailed sequence comparisons [34], there is a need to develop alternative bioinformatic workflows for genomic scale analyses. We suggest that a pluralistic approach integrating parametric approaches (e.g. [35]), network (protein similarity and derived genome network — e.g. [2, 11, 21, 36]), domain based approaches (e.g. [37]) and phylogenomics (e.g. [31, 38••] possibly including alignment free approaches [33^•^]) will be required to investigate the role of LGTs synthetically across eukaryotic taxonomic and proteome structural diversity.

## Conclusions

Based on LGT identified for microbial parasites, and an increasing number of free-living species, it is becoming apparent that LGT is a relevant process influencing the evolution of the coding capacity of eukaryotic genomes [39•, 40•], including those of multicellular forms [40•, 41]. Ancient (mitochondria) and more recent (e.g. primary and secondary plastids) EGT, combined with LGT from various bacterial sources have all influenced the pool of eukaryotic genes of bacterial origin. One challenge is to devise bioinformatic workflows to efficiently exploit the exponentially growing genome database and generate a global synthesis of the relative importance of EGT and LGT in shaping eukaryotic proteomes. Moreover, it is now clear that no pathway is safe from LGT, although negative selection may mean that replacements are less easily fixed and hence rarer in some pathways than others. A striking example is the paucity of LGT affecting the essential FeS cluster biosynthesis machinery. The great majority of eukaryotes posses a nuclear-encoded mitochondrial iron-sulfur cluster (ISC) system descended from the mitochondrial endosymbiont [42]. Nevertheless, LGTs from different prokaryotes to the common ancestor of *Entamoeba* and *Mastigamoeba* [43^•^] and independently to *Pygsuia* [44^•^], have replaced otherwise highly conserved components of the mitochondrial ISC machinery.

## References and recommended reading

Papers of particular interest, published within the period of review, have been highlighted as:

• of special interest
•• of outstanding interest

## Figures and Tables

**Table 1:** Variation of reported cases of LGT between species in a given study or between different studies for a given species for a selection of microbial parasites.

Species name	Higher rank taxonomy^a^	Total LGT count (%Proteome)^b^	P → E LGT^c^	E → E LGT^d^	Other LGT^e^	Methodology^f^	Reference
*Entamoeba histolytica*	Amoebozoa (Archamoebae)	199 (2.1% – 9090?)	197	NR	2 (virus)	Blast & Phylogeny	[47]
Entamoeba histolytica*	Amoebozoa (Archamoebae)	63 (0.68% – 9090)	51	12	NR	Blast & Phylogeny	[12^•^]
*Entamoeba dispar*	Amoebozoa (Archamoebae)	195 (1.90% – 10,262?)	194	NR	1 (virus)	Blast & Phylogeny	[47]
*Trichomonas vaginalis*	Excavata (Metamonada)	149 (0.24% – 59,681)	134	15	NR	Blast & Phylogeny	[12^•^]
*Giardia lamblia*	Excavata (Metamonada)	21 (0.36% – 6394)	15	6	NR	Blast & Phylogeny	[12^•^]
*Leishmania major*	Excavata (Discoba)	68 (0.96% – 7111)	63	5	NR	Blast & Phylogeny	[12^•^]
*Trypanosoma brucei*	Excavata (Discoba)	46 (0.47% – 9750)	45	1	NR	Blast & Phylogeny	[12^•^]
*Plasmodium falciparum*	SAR (Alveolata)	19 (0.36% – 5258)	18	1	NR	Blast & Phylogeny	[12^•^]
*Encephalitozoon cuniculi*	Opisthokonta (Nucletmycea)	3 (0.16 – 1918)	1	2	NR	Blast & Phylogeny	[12^•^]

a According to [15]. The two highest taxonomic ranks are indicated. SAR stands for the Stramenopiles, Alveolata and Rhizaria group.

b Values in brackets represent the fraction of LGT in % of the number of annotated protein coding genes, total is indicated after the dash. A question mark indicates the ambiguity about the exact dataset analysed as different annotations exist for a given genome.

c Candidate prokaryote to eukaryote LGTs. The great majority of candidates LGTs are from Bacteria.

d Candidate Eukaryote to Eukaryote LGTs.

e Additional sources of LGT investigated.

f Different criteria (BlastP and phylogenies) were used to select candidate LGT.

* Same dataset analysed in different publications — only two recent publications for one species were considered here. See [12^•^] for additional examples. NR: none reported.

**Table 2:** Transcription level of selected candidate LGTs of bacterial origins in *Trichomonas vaginalis*.

Locus tag	Annotation	Mean transcription^a^	Median transcription	Standard deviation
A recent LGT from a Firmicutes — [25]				
TVAG_243570 to TVAG_243830	Various — bacterial genomic segment with 27 annotated genes	6.7 (*n* = 27 genes, 11 conditions)	3.3 (*n* = 27)	9.0 (*n* = 27)
Bacterial LGT encoding candidate glycan degradation enzymes [12^•^]				
TVAG_010780	beta-N-acetylhexosaminidase — EC 3.2.1.52	585.6	607.2	326.1
TVAG_044970	N-acetylneuraminate lyase — EC 4.1.3.3	471.3	461.0	157.1
TVAG_123020^b^	alpha-mannosidase — EC 3.2.1.24	0.0	0.0	0.0
TVAG_371840^c^	alpha-mannosidase — EC 3.2.1.24	796.8	787.7	514.5
TVAG_270790	Acylglucosamine 2-epimerase — EC 5.1.3.8	1343.8	1400.7	465.1
TVAG_365600	beta-galactosidase — EC 3.2.1.23	626.6	620.9	251.1
TVAG_443530	alpha-fucosidase — EC 3.2.1.51	37.0	29.8	32.7
TVAG_483760	Beta-mannosidase — EC 3.2.1.25	178.3	132.7	167.7
TVAG_499550	Exo-alpha-sialidse — EC 3.2.1.18	687.5	528.7	483.3
TVAG_593180^d^	Glucosylceramidase — EC 3.2.1.45	0.0	0.0	0.0
Overall expression level [30]				
All 59681 annotated genes	NA	254.1	0.15	2662.6
All 33157 expressed genes >0 reads	NA	457.3	26.2	3559.2
All 20304 expressed genes ≥10 reads	NA	745.5	90.55	4524.8

a Transcriptomics data are from Gould *et al*. (2013) [30] with shown values being the mean normalised 3′end reads from the 11 distinct growth conditions investigated.

b Probable pseudogene: TVAG_123020 and TVAG_123030 are both annotated as alpha-mannosidase and a BlastX using the genomic sequence overlapping both coding sequences (locus DS113221, 216.7 kbp: 205,844–209,198) recovers one likely full-length *T. vaginalis* protein (TVAG_371840) and bacterial protein hits with two distinct reading frames (+2 and +3).

c Likely functional homologue to TVAG_123020 (55%ID to TVAG_123020). The result of a BlastP search is consistent with TVAG_123020 representing a bacterial LGT.

d Encoded by a small scaffold, hence it could represent a contaminant — locus DS145301, 1043 bp.
