# Supplementary material for: Lateral gene transfers and the origins of the eukaryote proteome: a view from microbial parasites
Source: Curr Opin Microbiol. 2015 Feb;23:155–62. doi: 10.1016/j.mib.2014.11.018 (PMC4728198; doi:10.1016/j.mib.2014.11.018)
Supplement: Supplementary file 1 [file mmc1.docx]

**Table S1. Variation of reported cases of LGT between species in a given study and between different studies for a given species for a selection of free-living microbial eukaryotes.**

| **Species name** | **Higher rank taxonomy^a^** | **Total LGT count (%Proteome)^b^** | **P->E LGT^c^** | **E->E LGT^d^** | **Other LGT^e^** | **Methodology^f^** | **Reference** |
| --- | --- | --- | --- | --- | --- | --- | --- |
| *Acanthamoeba castellanii*^g^ | Amoebozoa  (Discosea) | 450 (2.9% - 15,445) | 439 | NR | 11 (virus) | Blast & Phylogeny | [[1](#_ENREF_1)] |
| *Dictyostelium discoideum* | Amoebozoa  (Dictyostelia) | 61 (0.46% - 13,605) | 60 | 1 | NR | Blast & Phylogeny | [[2](#_ENREF_2)] |
| *Dictyostelium discoideum** | Amoebozoa  (Dictyostelia) | 92 (0.76%) | 89 | NR | 3 (virus) | Blast & Phylogeny | [[1](#_ENREF_1)] |
| *Naegleria gruberi* | Excavata  (Discoba) | 431 (2.7% - 15,727) | 427 | NR | 4 (virus) | Blast & Phylogeny | [[1](#_ENREF_1)] |
| *Naegleria gruberi** | Excavata  (Discoba) | 45 (0.29%) | 45 | NR | NR | Blast & Phylogeny, prok. hits only | [[3](#_ENREF_3)] |
| *Monosiga brevicolis* | Opisthokonta (Holozoa) | 405 (4.4% - 9,200) | 139 | 240 (algae) | 26 (bacteria or algae) | PhyloGenie, AlienG, and Darkhorse | [[4](#_ENREF_4)] |
| *Galdieria sulphuraria* | Archaeplastida (Rhodophyceae) | 75 (1.1% - 6623) | 73 | NR | 2 (virus) | Blast & Phylogeny | [[5](#_ENREF_5)] |

^a^According to [[6](#_ENREF_6)]. Only the two highest taxonomic ranks are indicated.

^b^Values in brackets represent the fraction of LGT in % of the number of annotated protein coding genes, total is indicated after the dash.

^c^Candidate prokaryote to eukaryote LGTs. The great majority of candidate LGTs are from Bacteria.

^d^Candidate Eukaryote to Eukaryote LGTs.

^e^Additional sources of LGT investigated.

^f^Different methologies and criteria were used to select candidate LGT, including variation in BlastP and phylogeny based approaches.

^g^*Acanthamoeba castellani* is a free-living species that occasionally infects humans and can cause various conditions in the skin, eyes, lungs or the brain [[7](#_ENREF_7)].

*Same dataset analysed in different publications. Only a selection of relevant publications is indicated.

NR: none reported.

**References**

1. Clarke M, Lohan AJ, Liu B, Lagkouvardos I, Roy S, Zafar N, Bertelli C, Schilde C, Kianianmomeni A, Burglin TR, et al.: **Genome of A*canthamoeba castellanii* highlights extensive lateral gene transfer and early evolution of tyrosine kinase signaling**. *Genome Biol* 2013, **14**:R11.

2. Alsmark C, Foster PG, Sicheritz-Ponten T, Nakjang S, Martin Embley T, Hirt RP: **Patterns of prokaryotic lateral gene transfers affecting parasitic microbial eukaryotes**. *Genome Biol* 2013, **14**:R19.

3. Fritz-Laylin LK, Prochnik SE, Ginger ML, Dacks JB, Carpenter ML, Field MC, Kuo A, Paredez A, Chapman J, Pham J, et al.: **The genome of *Naegleria gruberi* illuminates early eukaryotic versatility**. *Cell* 2010, **140**:631-642.

4. Yue J, Sun G, Hu X, Huang J: **The scale and evolutionary significance of horizontal gene transfer in the choanoflagellate *Monosiga brevicollis***. *BMC Genomics* 2013, **14**:729.

5. Schonknecht G, Chen WH, Ternes CM, Barbier GG, Shrestha RP, Stanke M, Brautigam A, Baker BJ, Banfield JF, Garavito RM, et al.: **Gene transfer from bacteria and archaea facilitated evolution of an extremophilic eukaryote**. *Science* 2013, **339**:1207-1210.

6. Adl SM, Simpson AG, Lane CE, Lukes J, Bass D, Bowser SS, Brown MW, Burki F, Dunthorn M, Hampl V, et al.: **The revised classification of eukaryotes**. *J Eukaryot Microbiol* 2012, **59**:429-493.

7. Siddiqui R, Khan NA: **Biology and pathogenesis of Acanthamoeba**. *Parasites & vectors* 2012, **5**:6.
